# Synergistic Effects of Anti-echinococcosis Drug Candidates Combined With Atovaquone in Culture Assays and Mice With Primary Infections of Echinococcus multilocularis

**DOI:** 10.7759/cureus.74324

**Published:** 2024-11-23

**Authors:** Hirokazu Kouguchi, Masahito Hidaka, Hiroyuki Matsuyama, Naoki Hayashi, Tomohito Koyano, Ryo Nakao, Nariaki Nonaka, Kinpei Yagi, Shigehiro Enkai

**Affiliations:** 1 Department of Infectious Diseases, Hokkaido Institute of Public Health, Sapporo, JPN; 2 Department of Disease Control, Laboratory of Parasitology, Graduate School of Infectious Diseases, Faculty of Veterinary Medicine, Hokkaido University, Sapporo, JPN; 3 Department of Gastroenterological Surgery, Graduate School of Medicine, Hokkaido University, Sapporo, JPN; 4 Department of Veterinary Research, Division of Parasitology, International Institute for Zoonosis Control, Hokkaido University, Sapporo, JPN; 5 Department of Pediatrics, Teikyo University School of Medicine, Tokyo, JPN; 6 School of Tropical Medicine and Global Health, Nagasaki University, Nagasaki, JPN

**Keywords:** 3-bromopyruvic acid, atovaquone, echinococcosis, echinococcus multilocularis, verapamil

## Abstract

Background

Alveolar echinococcosis (AE) is a fatal zoonotic disease distributed mainly in the Northern Hemisphere. At present, its curative treatment relies on surgery, and the development of effective drugs is needed. We previously demonstrated the anti-echinococcal effect of atovaquone (ATV) as a mitochondrial complex III inhibitor in both in vitro and in vivo experiments. However, the anti-echinococcal effect of ATV in vivo was limited, since ATV inhibits only aerobic respiration. In this study, we investigated whether ATV exhibits a synergistic effect when used in combination with other anti-echinococcal drug candidates, including mefloquine (MF), 3-bromopyruvic acid (3BP), crocin, and verapamil (Ver), thereby enhancing their antiparasitic effectiveness.

Methods

The synergistic effect of anti-echinococcal drug candidates with ATV was examined in culture experiments with *Echinococcus multilocularis* protoscoleces. Based on the results of these culture experiments, ATV and 3BP were individually and in combination orally administered to BALB/c mice infected with *E. multilocularis* (dose of 300 eggs). Each drug treatment was started three days prior to infection and continued until day 28 after egg administration, and the number of cysts located in the liver was evaluated (Experiment A). The anti-echinococcal effectiveness of the combination of ATV and 3BP was also evaluated by treating mice with *E. multilocularis* primary infection for eight weeks (Experiment B) and comparing the effects on cyst growth to those of albendazole (ABZ).

Results

Culture experiments with *E. multilocularis* protoscoleces showed that the combined treatments of ATV with 3BP, MF, and Ver were more effective at parasite elimination under both aerobic and anaerobic conditions than the single drug treatments. Crocin was ineffective in the culture assay. In Experiment A, the number of cysts was significantly reduced only in the groups treated with ABZ alone (median 48.0, interquartile range 36.3-58.0) and the combination of ATV and 3BP (median 54.5, interquartile range 46.5-62.8) compared to the control (median 90.0, interquartile range 67.0-100.5). MF and Ver did not exhibit significant in vivo effects on their own. In Experiment B, the group treated with ATV + 3BP showed a similar anti-echinococcal effect as the group treated with ABZ alone.

Conclusion

In the culture assay, ATV in combination with 3BP, MF, and Ver showed a synergistic effect, enhancing the anti-echinococcal effect under both aerobic and anaerobic conditions. In mice experimentally treated with primary hydatid cysts, co-administration of ATV and 3BP showed a significant preventive effect against infection and demonstrated therapeutic efficacy comparable to that of ABZ. These findings are anticipated to contribute to the development of more effective therapeutic agents for AE.

## Introduction

Alveolar echinococcosis (AE) is a fatal zoonotic disease caused by the larval stage of *Echinococcus multilocularis*, leading to significant health issues and economic losses globally. Approximately 18,000 new human cases are reported annually in the Northern Hemisphere [1]. In humans, AE results from the accidental oral ingestion of parasite eggs excreted by foxes as the definitive host. Once inside the human body, the larvae grow into tumor-like masses in the liver. The condition can remain asymptomatic for many years, and surgical removal of affected areas can be difficult by the time it is detected. Long-term administration of albendazole (ABZ) is necessary if lesions cannot be completely excised. While ABZ is currently the only effective medication against AE, it does not completely eliminate the disease. Furthermore, serious side effects have been reported with long-term use. For example, about 16% of patients experience side effects such as hepatotoxicity with benzimidazole therapy (which includes ABZ) [2,3]. Consequently, the development of new treatments and therapeutics is needed. Some reports have demonstrated the anti-echinococcal effects of several chemical compounds in experimentally infected mice [4]. Mefloquine (MF), an anti-malaria drug, has been reported to be effective against *E. multilocularis* in both in vitro and in vivo studies [5,6]. The potential of 3-bromopyruvic acid (3BP), a hexokinase inhibitor, for anti-echinococcal activity via inhibiting the glycolytic pathway was investigated. Xin et al. reported that 3BP showed significant efficacy in the treatment of mice infected with *E. multilocularis* when administered at a dose of 25 mg/kg twice a week [7]. Crocin is a water-soluble carotenoid that primarily exerts its anti-tumor effects by inhibiting the expression of matrix metalloproteins. This compound has also been reported to possess anti-echinococcal activity [8]. Verapamil (Ver) is a compound that exhibits calcium channel-blocking properties and is widely used in clinical settings as an anti-arrhythmic medication. Calcium channels have shown promise as a drug target for anti-echinococcal agents, and indeed, anti-echinococcal activity has been reported in both in vitro and in vivo studies [9].

These studies provide valuable data for the development of anti-echinococcal drugs. However, except for the report on MF [5,6], they employed in vivo tests using secondary hydatid cysts, which are infections caused by transplantation or intravenous injection of protoscoleces (as an artificial experimental infection). Our group has been investigating the efficacy of drugs using primary hydatid cysts, which produce lesions in the livers of mice through oral infection with parasite eggs, closely simulating an actual infection [10,11].

We have revealed that *E. multilocularis* can utilize two different respiratory methods to produce adenosine triphosphate (ATP), depending on the oxygen availability in its host's environment [10]. In the absence of oxygen, the parasite employs fumarate respiration, using fumarate in an anaerobic respiratory chain. Conversely, when oxygen is abundant, it switches to oxidative phosphorylation within the aerobic respiratory chain. This ability to alternate respiratory chains enables *E. multilocularis* to adapt to varying oxygen conditions and ensures its survival within the host. Atovaquone (ATV) is known as a safe antimalarial drug in clinical practice. We have reported that ATV displays anti-echinococcal effects both in vitro and in vivo [10]. The effects of ATV are attributed to its strong inhibition of complex III of the aerobic respiratory chain within mitochondria. However, the therapeutic effect in infected mice was limited because ATV does not inhibit the anaerobic respiratory chain. Notably, when ATV is used in combination with ABZ, it shows enhanced efficacy, indicating potential synergistic effects [12]. ATV may synergistically enhance the potency of compounds that do not have sufficient anti-echinococcal effects on their own. Therefore, we tested ATV in combination with MF, 3BP, crocin, and Ver for possible synergistic effects against *E. multilocularis* protoscoleces in culture experiments. Based on the result, the combination of ATV and 3BP was examined for its anti-echinococcal effect on experimentally infected mice with *E. multilocularis* eggs.

## Materials and methods

*E. multilocularis* (Nemuro strain), which is maintained at the Hokkaido Institute of Public Health, Sapporo, Japan, was used. Cotton rats (*Sigmodon hispidus*) were experimentally infected by orally inoculating 200 *E. multilocularis* eggs and reared for at least four months to obtain mature protoscoleces. Infected rats were sacrificed after an isoflurane overdose to obtain cyst tissues containing *E. multilocularis* protoscoleces. To isolate protoscoleces, the cyst tissue was minced with scissors, passed through a metal mesh to completely shred it, and then repeatedly suspended and washed with phosphate-buffered saline (PBS) containing penicillin (1,000,000 units/L) and streptomycin (1 g/L) in an 18 cm glass test tube, a method based on buoyancy differences between protoscoleces and other tissues. Isolated protoscoleces were transferred to glass petri dishes and gently rotated in PBS containing penicillin (1,000,000 units/L) and streptomycin (1 g/L) to remove calcareous corpuscles [10,13].

Culture assays using *E. multilocularis* protoscoleces

Culture assays were performed as previously reported [10,13]. ATV (Tokyo Chemical Industry Co., Ltd., Tokyo, Japan), MF hydrochloride (Tokyo Chemical Industry Co., Ltd), 3BP acid (Sigma-Aldrich, St. Louis, MO, USA), crocin (PhytoLab Inc., Troy, MI, USA), and Ver hydrochloride (FUJIFILM Wako Pure Chemical Corp., Kobe, Japan) were purchased and used in this study. Drug candidates were added on the day that the protoscoleces culture was initiated. To assess the efficacy of drug candidates against *E. multilocularis* protoscoleces, the parasites were treated with ATV, MF, 3BP, crocin, and Ver individually and in combination at a final concentration of 50 μM each in the culture medium. Rotenone was used as the control for comparison with previous results [10]. Ver was added at a final concentration of 50 μg/mL, based on the concentrations reported previously [9]. More than 120 protoscoleces were removed daily from the 24-well plates, stained with trypan blue for 30 minutes, and then counted. Viability was calculated as the number of dead protoscoleces that did not excrete trypan blue. These assays were conducted in triplicate. The day of incubation was set as day 0, and the assay was continued for seven days.

Animal experiment

This study was performed in strict accordance with the National Institutes of Health Guide for the Care and Use of Laboratory Animals, and the Ethics Committee of the Hokkaido Institute of Public Health approved the animal experiment protocol (permit number: K22-1/K23-7). All surgeries were performed under sodium pentobarbital anesthesia, and every effort was made to minimize the suffering of animals.

Examination of drug efficacy using experimentally-infected mice

BALB/c mice (female, six to seven weeks old) were purchased from Sankyo Lab Service Corp., Tokyo, Japan, and acclimatized for one week. Parasite eggs were purified from the feces of dogs experimentally infected with *E. multilocularis*, according to a previous report [14].

Experiment A

ATV, MF, 3BP, and Ver, which were selected based on culture assays, were used. Preliminary experiments confirmed that BALB/c mice ingest approximately 3 g of experimental animal diet (CMF; Oriental Yeast Co., Ltd., Osaka, Japan) per day. Each compound was blended with the pulverized feed using a Waring blender until a uniform consistency was reached, according to a previous report [10]. ATV was prepared so that the dosage was approximately 200 mg/kg/day. Specifically, 2400 mg of ATV was mixed with 1.8 kg of crushed experimental animal diet, molded using a milling machine, and dried at 50°C overnight. MF was prepared with a dosage of 25 mg/kg/day. In other words, 300 mg of MF was added to 1.8 kg of crushed feed to create a diet containing MF, in the same manner as described above. Diets containing ATV and MF were prepared by adding 2400 mg of ATV and 300 mg of MF to 1.8 kg of crushed feed and drying it at 50°C overnight. 3BP was administered with honey twice a week at 25 mg/kg, according to a previous report [7]. The ATV + 3BP group was fed the aforementioned ATV feed, and 3BP was administered with honey twice a week at a dose of 25 mg/kg. Ver was prepared to provide 40 mg/kg/day. The diet containing ATV + Ver was prepared by adding 2400 mg of ATV and 480 mg of Ver to 1.8 kg of crushed feed. In the ABZ treatment, 2400 mg of ABZ was mixed with 1.8 kg of crushed feed. It was confirmed that MF and Ver were not inactivated by heating at 50°C overnight, and a similar survival rate of protoscoleces was observed in the culture assay (data not shown). A total of 88 BALB/c mice (female, seven weeks old) were divided into nine groups as follows: control (10 mice), ATV (10 mice), MF (10 mice), 3BP (10 mice), Ver (10 mice), MF + ATV (10 mice), ATV + 3BP (10 mice), ATV + Ver (10 mice), and ABZ (eight mice) groups. Three days prior to infection, mice were orally administered each drug candidate by ad libitum feeding of the drug-mixed feed, and administration of each drug candidate continued for four weeks. The control group consumed untreated feed. All animals had access to water ad libitum. In this experiment, 300 eggs, prepared from the feces of an *E. multilocularis*-infected dog, were administered orally. All mice were sacrificed four weeks after infection, and necropsies were performed. The number of AE cysts on liver sections (sliced to 1 mm with a scalpel) was counted using a head loupe.

Experiment B

**Figure 1:**
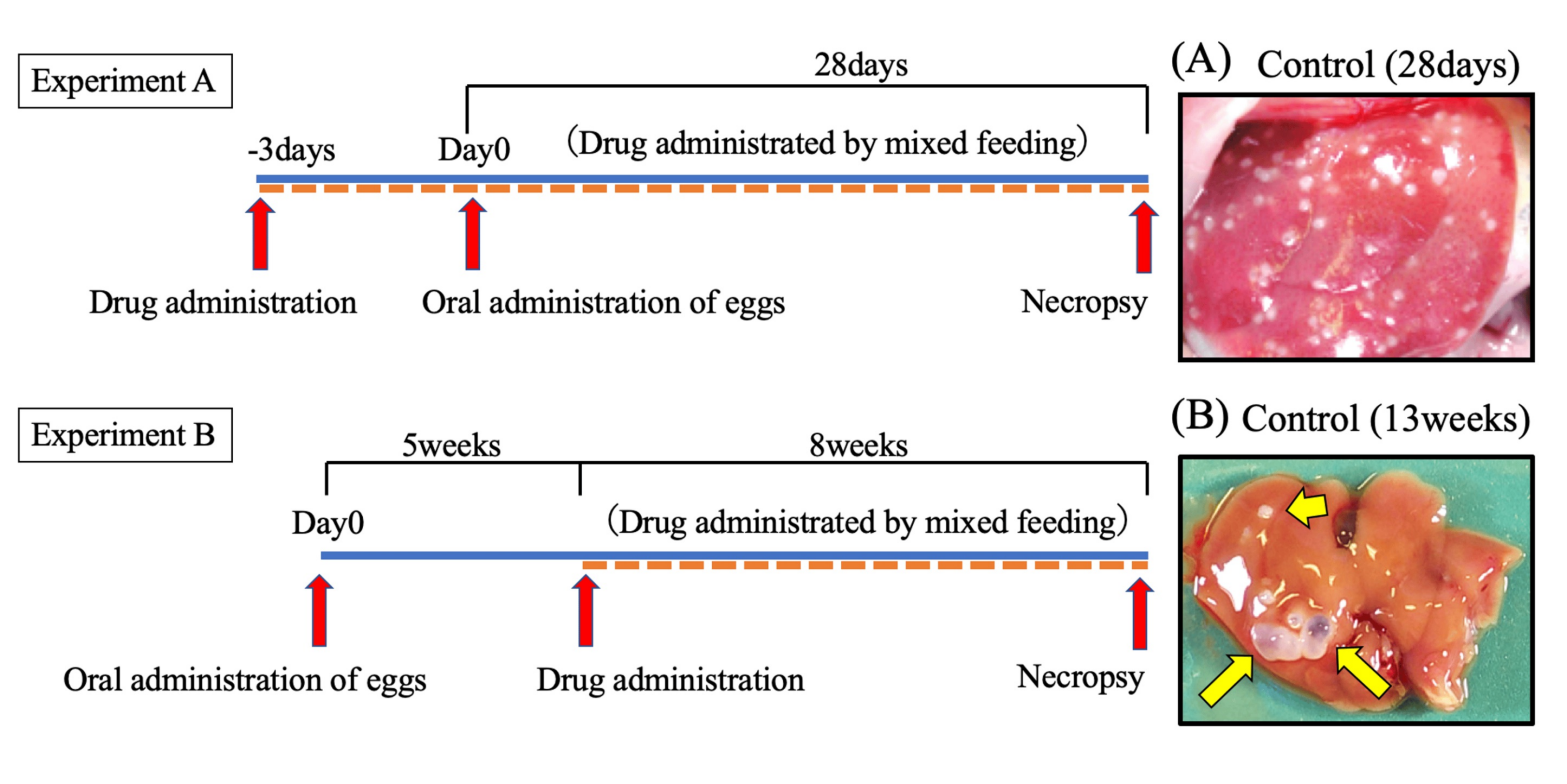
In vivo experiment protocol Overview of the experimental flow of the anti-echinococcal effect of drug candidates using mice with primary *E. multilocularis* infection. BALB/c mice were orally administered 300 eggs prepared from the feces of an *E. multilocularis*-infected dog (Experiment A, n = 10; Experiment B, n = 5). The images show typical cysts observed in an untreated mouse liver. (A) Cysts appear as small white dots on the liver. (B) Cysts are indicated by arrows. *E. multilocularis*, *Echinococcus multilocularis*

Administration of ATV + 3BP was compared with the control and ABZ groups to determine its therapeutic effect in reducing cyst size. BALB/c mice (female, six to seven weeks old, average body weight of 25 g, and daily average food intake of 3.0 g) were infected orally with 300 parasite eggs in a 400-μL suspension prepared from the feces of an *E. multilocularis*-infected dog. At four weeks post-infection, three mice were necropsied to confirm the presence of cysts (1-2 mm diameter, 1-2 mm long) in the liver. After confirming cyst formation, mice were randomly allocated into three groups of six animals each, as follows: ATV + 3BP, ABZ, and control groups. ATV + 3BP and ABZ were fed in the same manner as Experiment A, respectively, and untreated mice were used as controls. The mice were subjected to the treatments for 12 weeks by feeding the drug-mixed feed ad libitum. Necropsy was performed at the end of drug administration, and the proportion of cysts on the liver surface was measured using digital image analysis software (ImageJ, Bethesda, MD, USA) to evaluate cyst growth. Experiment A and Experiment B are represented in Figure 1.

Statistical analysis

In Experiment A, Dunnett's multiple comparison test was used to determine differences in the number of *E. multilocularis* cysts between the control and each drug candidate treatment. The analyses were performed using the MASS and multcomp packages in R version 4.1.3 (R Foundation for Statistical Computing, Vienna, Austria) [15]. For Experiment B, Steel-Dwass's multiple comparison test was used to determine differences in cyst area between each drug candidate treatment. The analyses were performed using R version 4.1.3 [15].

## Results

Culture treatment assay

**Figure 2:**
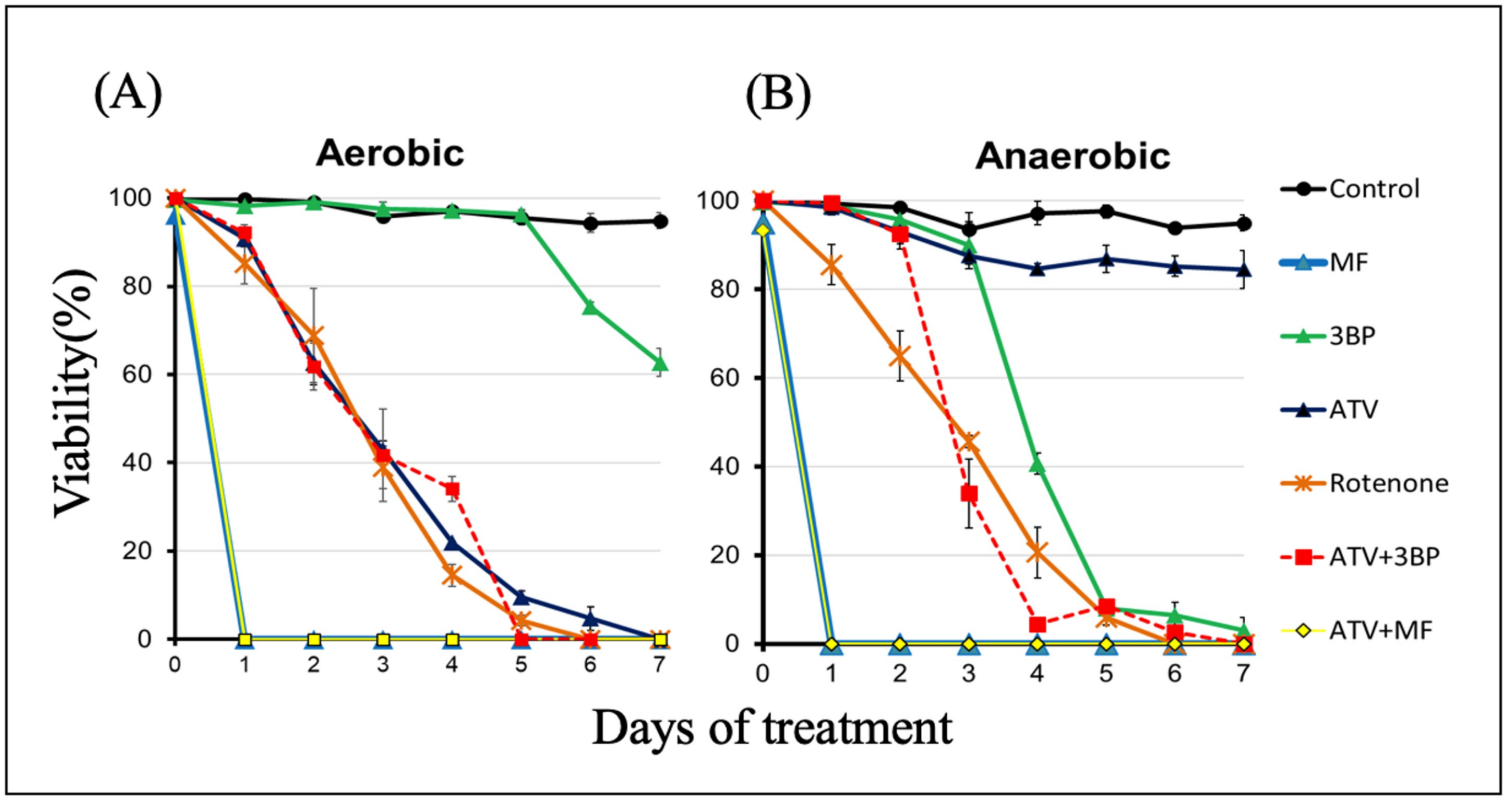
Culture assays Culture assays under aerobic (A) and anaerobic (B) conditions (oxygen concentration < 0.3%). *E. multilocularis* protoscoleces were treated with ATV, MF, 3BP, and their combinations at a final concentration of 50 μM in each medium. Protoscoleces survival was evaluated by the ability to eliminate trypan blue. Protoscoleces were counted in triplicate from day 0 to day 7. Statistical analysis was performed on mean survival (%), and the standard deviation of triplicate samples is indicated by error bars. Rotenone was used as a positive control. The data in this figure were cited from our previous study [13]. ATV, atovaquone; MF, mefloquine; 3BP, 3-bromopyruvate; *E. multilocularis*, *Echinococcus multilocularis*

Figure 2A presents our previously published data [13]. That is, aerobic culture experiments with *E. multilocularis* protoscoleces show the antiparasitic effects of 50 μM ATV, rotenone, MF, 3BP, and a combination of ATV and two other drugs. Rotenone was used as the positive control, as previously reported [10]. ATV eliminated all protoscoleces on day 7, as previously reported, but the addition of MF alone or in combination with ATV (ATV + MF) quickly killed all protoscoleces on day 1. The addition of 3BP alone killed 37% of protoscoleces on day 7, and ATV + 3BP reduced viability to the same rate as ATV alone; however, the combination of ATV + 3BP eliminated all protoscoleces on day 5, two days earlier than ATV alone. Figure 2B shows the results of the culture assay under anaerobic conditions. ATV showed almost no anti-echinococcal effect on the protoscoleces, as previously reported [10]. MF alone and in combination (ATV + MF) killed all protoscoleces on day 1, as well as under aerobic conditions. 3BP killed more than 90% of the protoscoleces on day 5, and the effect was more pronounced than under aerobic conditions. The combination of ATV and 3BP killed all protoscoleces on day 7 under anaerobic conditions, with a more rapid decline in viability than that of 3BP alone.

**Figure 3:**
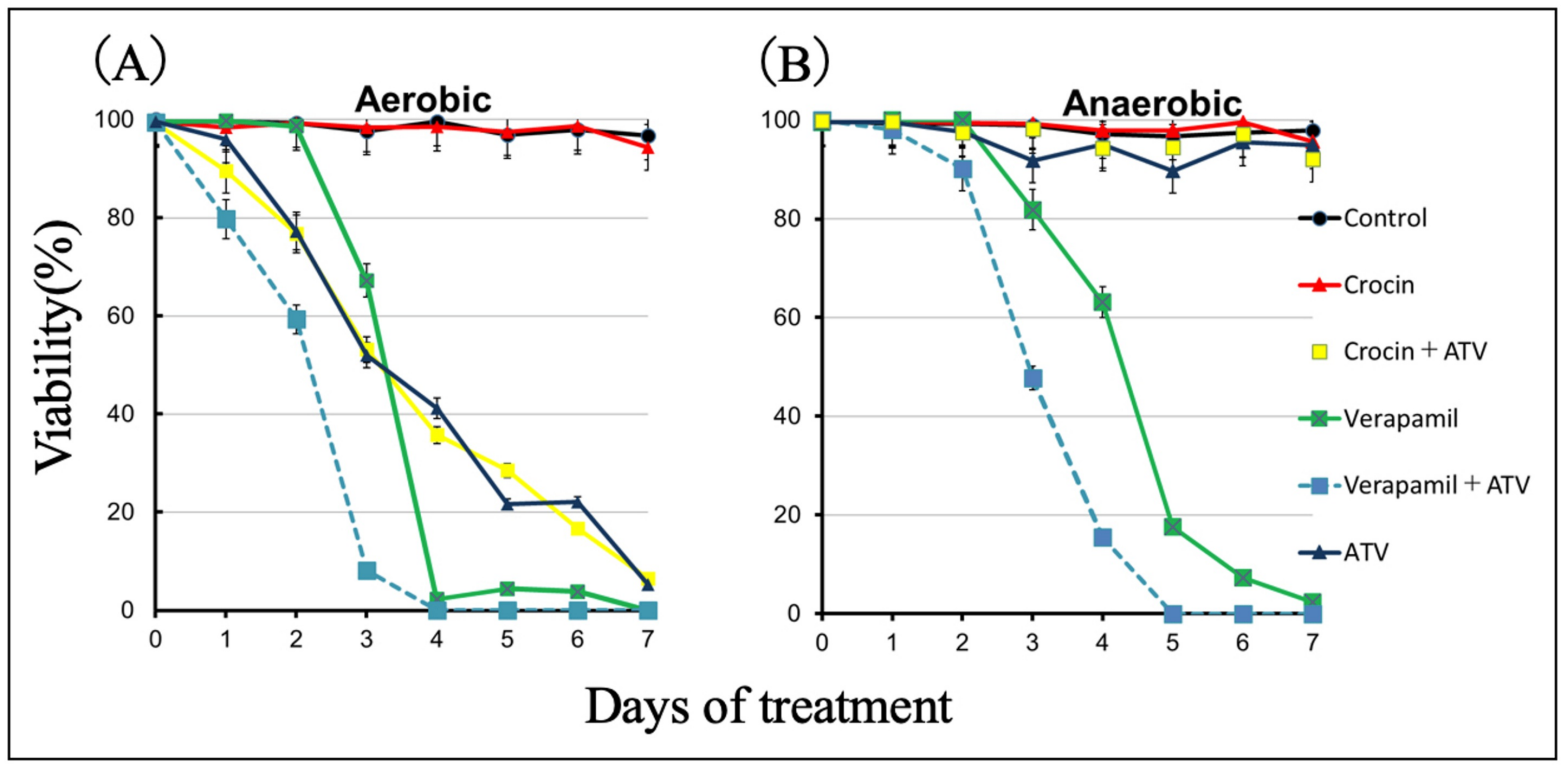
Culture assays Culture assays under aerobic (A) and anaerobic (B) conditions (oxygen concentration < 0.3%). Protoscoleces of *E. multilocularis *were treated with ATV, crocin, Ver, and their combinations. Crocin and ATV were treated at a final concentration of 50 µM each in the medium. The final concentration of Ver alone in the medium was 50 μg/mL. The combination of ATV and Ver was set at 50 μM ATV and 50 μg/mL Ver. Survival of protoscoleces was assessed by the ability to eliminate trypan blue. Protoscoleces were counted in triplicate from day 0 to day 7. Statistical analysis was performed on mean survival (%), and the standard deviation of triplicate samples is indicated by error bars. ATV, atovaquone; Ver, verapamil; *E. multilocularis*, *Echinococcus multilocularis*

Figure 3A shows the antiparasitic effects of 50 µM ATV, crocin, Ver, and a combination of ATV and the two other drugs in an aerobic culture experiment using *E. multilocularis* protoscoleces. ATV killed all protoscoleces on day 7, which is consistent with previous reports [10], but crocin alone showed only about a 2% reduction in viability compared to the control. The combination of ATV + crocin showed a similar reduction in viability as ATV, with no synergistic effect. When Ver was added alone, all protoscoleces were killed on day 5. The combination of ATV and Ver killed 85% of protoscoleces on day 4, exhibiting a faster decrease in viability compared to ATV or Ver alone. Figure 3B shows the results of the culture assay under anaerobic conditions, where ATV showed little anti-echinococcal effect, as previously reported [10]. Crocin alone and in combination (ATV + crocin) did not show any mortality in the experimental system used in this study, as observed under aerobic conditions. Ver killed all protoscoleces on day 7, an effect similar to that under aerobic conditions; the combination of ATV and Ver killed all protoscoleces on day 6 under anaerobic conditions, and the decrease in survival appeared to be earlier than that of Ver alone.

Anti-echinococcal efficacy in mice orally infected with *E. multilocularis* eggs 

**Figure 4:**
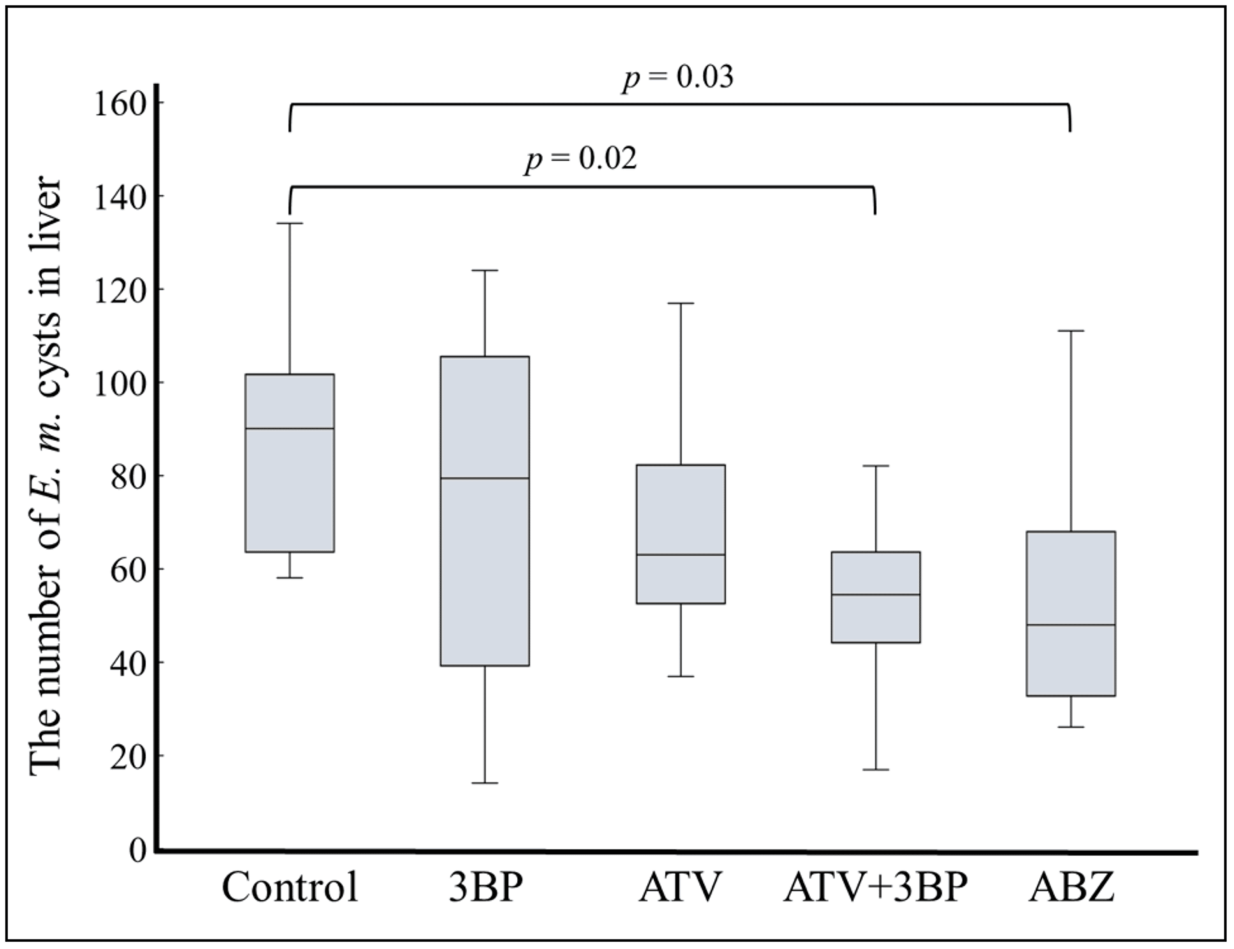
Animal experiment A ATV, 3BP, ATV + 3BP, or ABZ were administered by mixed feeding (200 mg/kg/day each) prior to infection with parasite eggs. 3BP was mixed with honey and administered at 25 mg/kg/day twice weekly. Subsequently, 300 parasite eggs prepared from the feces of an *E. multilocularis*-infected dog were orally administered to BALB/C (n = 10) mice. Administration of each drug was continued until day 28. As shown in the figure, only the ATV + 3BP and ABZ groups showed significant reductions in cyst counts; notably, the ATV + 3BP mixture was more effective than 3BP or ATV alone. MF alone, Ver alone, and their combinations with ATV showed no significant anti-echinococcal effect in this experiment (data not shown). ATV, atovaquone; 3BP, 3-bromopyruvate; ABZ, albendazole; MF, mefloquine; Ver, verapamil; *E. multilocularis*, *Echinococcus multilocularis*

**Figure 5:**
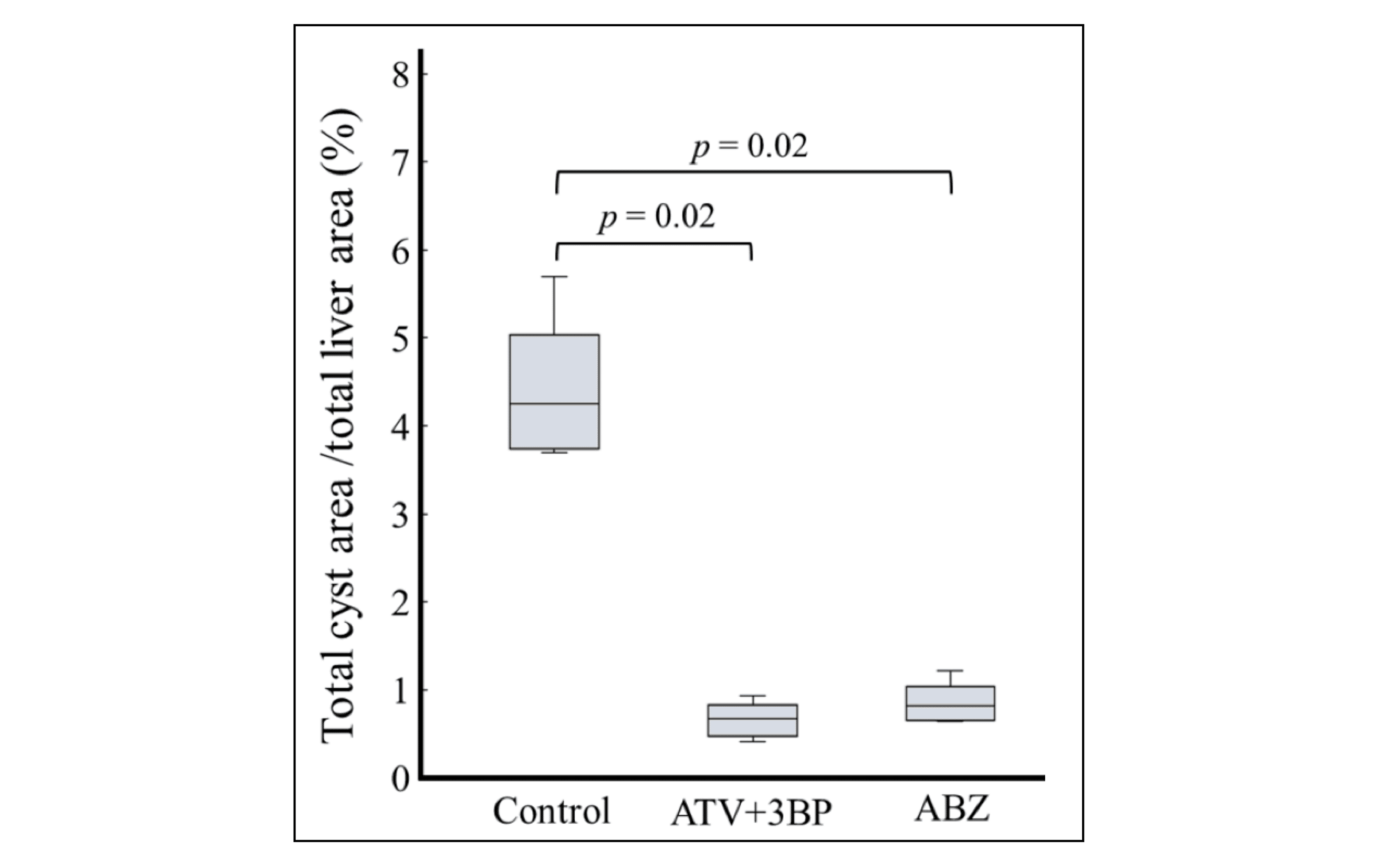
Animal experiment B BALB/c mice (n = 6) were orally administered 300 parasite eggs prepared from an *E. multilocularis*-infected dog. Five weeks later, ATV + 3BP or ABZ was administered by mixed feeding (200 mg/kg/day each). 3BP was administered at 25 mg/kg/day mixed with honey twice a week. ATV + 3BP administration was as effective as ABZ. ATV, atovaquone; 3BP, 3-bromopyruvate; ABZ, albendazole; *E. multilocularis*, *Echinococcus multilocularis*

Figure 4 shows the results of Experiment A, where either ATV, 3BP, and ABZ alone, or ATV + 3BP was administered to *E. multilocularis*-infected mice. ABZ alone was added as a standard treatment control. In the present study, the MF, Ver, ATV + MF, and ATV + Ver groups did not show a significant reduction in cyst count (data not shown). Crocin showed no anti-echinococcal effect in the culture assay, so in vivo experiments were not performed in this study. Of all groups, statistically significant reductions in AE cyst counts compared to the control group were seen in the ATV + 3BP group and the ABZ group. While administration of 3BP or ATV alone showed lower cyst counts than the controls, this was not statistically significant. Hence, in this study, treatment with ABZ alone and ATV + 3BP was found to show statistically significant effects. In Experiment B, ATV + 3BP was administered to mice at five weeks post-infection for eight weeks, as shown in Figure 5. As a result, the size of foci on the liver surface in the ATV + 3BP group was reduced compared to that of the control group, and it was suppressed to within about 1%, similar to the results with ABZ.

## Discussion

It has been reported that ATV treatment significantly reduced the size of liver cysts in mice with primary alveolar hydatid disease, compared to untreated controls [10]. However, ATV was not fully effective, as it only inhibits aerobic respiration of the parasite, compared to ABZ treatment. Thus, there is a need for compounds that inhibit fumarate respiration under anaerobic conditions. On the other hand, we reported that the co-administration of ATV and ABZ had a synergistic effect in the AE treatment of mice [12]. In this study, we evaluated the synergistic effect of existing candidate drugs (MF, 3BP, crocin, and Ver) in combination with ATV.

3BP exhibited greater efficacy in killing protoscoleces under anaerobic compared to aerobic conditions (Figure 2). This suggests that the parasite is more dependent on glycolysis under anaerobic conditions. Co-administration of ATV and 3BP showed enhanced therapeutic efficacy compared to either ATV or 3BP alone (Figure 4). These results indicate that the protoscoleces depend on glycolysis for the production of energy and intermediates under both aerobic and anaerobic conditions [16,17]. Therefore, inhibition of the glycolytic pathway, which is essential for both aerobic and anaerobic respiration, would be an effective approach to targeting *E. multilocularis*. Focusing on anaerobic glycolysis by inhibiting enzymes such as hexokinase, glyceraldehyde 3-phosphate dehydrogenase (GAPDH), fructose bisphosphate aldolase, phosphoglycerate kinase, phosphoglyceromutase, and lactate dehydrogenase has the potential to reduce ATP production. Several compounds are recognized as inhibitors of the glycolytic pathway, including 3BP [18-20]. *E. multilocularis* possesses a complete set of glycolytic enzymes [21]. The inhibition of the rate-limiting enzyme in the glycolytic pathway appears to block all ATP biosynthetic pathways, irrespective of the presence of oxygen. However, 3BP exhibits reduced efficacy under aerobic and anaerobic conditions in the absence of ATV. 

Even in the presence of minimal oxygen as the anaerobic condition, there might exist pathways capable of donating electrons to the electron transport chain beyond the glycolytic system. It is possible that enzymes other than complexes I and II, such as G3PDH, electron-transferring-flavoprotein dehydrogenase (ETFDH), dihydroorotate dehydrogenase (DHOD), and proline dehydrogenase (PRODH), which supply electrons to quinone, exist upstream on the oxygen respiratory chain pathway [22]. G3PDH and ETFDH are membrane proteins located on the matrix side of the mitochondrial inner membrane that oxidize glycerol-3-phosphate (G3P) and electron transfer flavoprotein (ETF), respectively, which are obtained from gluconeogenesis and the lipid metabolism pathway, to reduce quinones [22]. DHODH and PRODH are located on the intermembrane space-facing side of the mitochondrial inner membrane, where they play roles in pyrimidine synthesis and proline metabolic pathways, respectively. Each enzyme catalyzes the oxidation of dihydroorotate to orotate and proline to pyrroline-5-carboxylate, respectively, which leads to the generation of quinol [22]. Although there are no reports of such enzyme activity measurements for *E. multilocularis*, these enzymes are highly conserved and functional in multicellular organisms. Therefore, even if inhibition of the glycolytic pathway stops the supply of electrons from complexes I and II, as long as there is a small amount of residual oxygen, the parasite can produce ATP through the electrons supplied by these enzymes. ATV may contribute to the inhibition of ATP production by strongly inhibiting complex III, thereby blocking the transfer of electrons supplied by these enzymes to the downstream complexes III and IV. In addition, the co-administration of 3BP and ATV demonstrated a prophylactic effect against infection. In the larval stage, the oncosphere, which migrates from the blood to the liver and other organs, may utilize both the electron transfer chain and the anaerobic glycolytic pathway, since ATV or 3BP alone exhibits no prophylactic effect. Consequently, the parasites exposed to these compounds within the vascular system fail to establish themselves in the hepatic parenchyma. It is noteworthy that the co-administration of ATV and 3BP demonstrated therapeutic and prophylactic effects in infected mice. 

Crocin is known as an anti-tumor drug that blocks the expression of matrix metalloproteinases. Previous research has reported that crocin exhibits antiparasitic effects in *E. multilocularis* [8]. However, in the present study, protoscoleces treated with crocin were observed to be motile until day 7, with very low mortality. The reason for this remains unclear, but differences in the cytotoxicity and quality of the reagents used in the previous report could be contributing factors. Indeed, in the above report, crocin was shown to exert cytotoxic effects in mammalian cells [8].

Besides ABZ (which has reported side effects), the chemical compounds tested in the current study offer a new option for the chemotherapeutic treatment of AE. However, the present study had some limitations. Altering the frequency and route of drug administration may lead to even better outcomes. Furthermore, as previously mentioned, the development of drug derivatives specific to AE could potentially yield superior results.

## Conclusions

In culture assays, ATV has been found to exhibit a synergistic effect when combined with 3BP, MF, and Ver, thereby enhancing the anti-echinococcal effects under both aerobic and anaerobic conditions. When ATV and 3BP were co-applied in a mouse model for the treatment of primary hydatid cysts, infection was obviously prevented, with high therapeutic efficacy comparable to ABZ. One possible explanation for the synergistic effect of ATV + 3BP is that, while 3BP inhibits hexokinase in the anaerobic glycolysis pathway, blocking the upstream energy production routes, ATV may have increased its lethal effects by inhibiting complex III, which would block the flow of electrons from downstream enzymes and fully halt the production of ATP in the oxygen respiratory chain pathway. These findings hold promise for the development of more potent therapeutic drugs.

## References

[1] Torgerson PR, Keller K, Magnotta M, Ragland N (2010). The global burden of alveolar echinococcosis. PLoS Negl Trop Dis.

[2] Wen H, Vuitton L, Tuxun T, Li J, Vuitton DA, Zhang W, McManus DP (2019). Echinococcosis: advances in the 21st century. Clin Microbiol Rev.

[3] Steiger U, Cotting J, Reichen J (1990). Albendazole treatment of echinococcosis in humans: effects on microsomal metabolism and drug tolerance. Clin Pharmacol Ther.

[4] Siles-Lucas M, Casulli A, Cirilli R, Carmena D (2018). Progress in the pharmacological treatment of human cystic and alveolar echinococcosis: compounds and therapeutic targets. PLoS Negl Trop Dis.

[5] Rufener R, Ritler D, Zielinski J (2018). Activity of mefloquine and mefloquine derivatives against Echinococcus multilocularis. Int J Parasitol Drugs Drug Resist.

[6] Lundström-Stadelmann B, Rufener R, Hemphill A (2020). Drug repurposing applied: activity of the anti-malarial mefloquine against Echinococcus multilocularis. Int J Parasitol Drugs Drug Resist.

[7] Xin Q, Yuan M, Li H, Song X, Lu J, Jing T (2019). In vitro and in vivo effects of 3-bromopyruvate against Echinococcus metacestodes. Vet Res.

[8] Liu C, Fan H, Guan L, Ge RL, Ma L (2021). In vivo and in vitro efficacy of crocin against Echinococcus multilocularis. Parasit Vectors.

[9] Gao HJ, Sun XD, Luo YP (2021). Anti-echinococcal effect of verapamil involving the regulation of the calcium/calmodulin-dependent protein kinase II response in vitro and in a murine infection model. Parasit Vectors.

[10] Enkai S, Inaoka DK, Kouguchi H, Irie T, Yagi K, Kita K (2020). Mitochondrial complex III in larval stage of Echinococcus multilocularis as a potential chemotherapeutic target and in vivo efficacy of atovaquone against primary hydatid cysts. Parasitol Int.

[11] Matsumoto J, Sakamoto K, Shinjyo N (2008). Anaerobic NADH-fumarate reductase system is predominant in the respiratory chain of Echinococcus multilocularis, providing a novel target for the chemotherapy of alveolar echinococcosis. Antimicrob Agents Chemother.

[12] Enkai S, Kouguchi H, Inaoka DK, Irie T, Yagi K, Kita K (2021). In vivo efficacy of combination therapy with albendazole and atovaquone against primary hydatid cysts in mice. Eur J Clin Microbiol Infect Dis.

[13] Kouguchi H, Enkai S, Matsuyama H, Hidaka M, Inaoka DK, Kita K, Yagi K (2022). Data on the combined effect of atovaquone, mefloquine, and 3-bromopyruvic acid against Echinococcus multilocularis protoscoleces. Data Brief.

[14] Kouguchi H, Irie T, Matsumoto J, Nakao R, Sugano Y, Oku Y, Yagi K (2016). The timing of worm exclusion in dogs repeatedly infected with the cestode Echinococcus multilocularis. J Helminthol.

[15] (2024). The R Project for statistical computing. https://www.r-project.org/.

[16] Agosin M (1957). Studies on the metabolism of Echinococcus granulosus. II. Some observations on the carbohydrate metabolism of hydatid cyst scolices. Exp Parasitol.

[17] McManus DP, Smyth JD (1978). Differences in the chemical composition and carbohydrate metabolism of Echinococcus granulosus (horse and sheep strains) and E. multilocularis. Parasitology.

[18] Ko YH, Pedersen PL, Geschwind JF (2001). Glucose catabolism in the rabbit VX2 tumor model for liver cancer: characterization and targeting hexokinase. Cancer Lett.

[19] Zhang Q, Zhang Y, Zhang P (2014). Hexokinase II inhibitor, 3-BrPA induced autophagy by stimulating ROS formation in human breast cancer cells. Genes Cancer.

[20] Xu R, Pelicano H, Zhou Y (2005). Inhibition of glycolysis in cancer cells: a novel strategy to overcome drug resistance associated with mitochondrial respiratory defect and hypoxia. Cancer Res.

[21] Tsai IJ, Zarowiecki M, Holroyd N (2013). The genomes of four tapeworm species reveal adaptations to parasitism. Nature.

[22] Banerjee R, Purhonen J, Kallijärvi J (2022). The mitochondrial coenzyme Q junction and complex III: biochemistry and pathophysiology. FEBS J.

